# Post transcriptional regulation of HIV-1 gene expression by MATR3, PSF and Rev

**DOI:** 10.1186/1742-4690-10-S1-P37

**Published:** 2013-09-19

**Authors:** Lavina Gharu, Ania Kula, Maryana Bardina, Alessandro Marcello

**Affiliations:** 1 Molecular Virology Department, International Centre for Genetic Engineering and Biotechnology (ICGEB), Trieste, Italy

## Background

HIV-1 gene expression and replication are regulated at several levels. Incompletely spliced viral RNAs and full length genomic RNA contain the RRE element and are bound by the viral *trans*-acting protein Rev to be transported out of the nucleus. Previously, we found, through a novel proteomic approach, that the nuclear matrix protein MATR3 is a cofactor of Rev mediated RNA export [Kula A *et al.: Retrovirology* 2011, **8**:60]. More recently we demonstrated that pleiotropic polypyrimidine tract binding protein associated splicing factor (PSF) binds viral RNA and is associated with MATR3 [Kula A., Gharu L, Marcello A: *Virology* 2013, **435**(2):329-340].

## Materials and methods

To investigate the functional role of MATR3 and PSF in HIV-1 replication and their involvement in Rev activity, we measured the effect of RNAi mediated MATR3 and PSF knock down in infected Jurkat cells. Quantitative RT PCR analysis was performed to measure the levels of unspliced and spliced mRNA transcripts in nuclear and cytoplasmic fractions. Co-immunoprecipitation experiments were done to explore the interactions between MATR3, PSF and HIV Rev protein. J-lat 8.4 cells were used as a model for HIV-1 latency. Resting CD4+ T lymphocytes were isolated from peripheral blood.

## Results

We showed that knock down of either of the two cellular factors MATR3 and PSF inhibited Rev activity specifically on unspliced HIV-1 RNAs. While MATR3 depletion suppressed the Rev dependent export of HIV-1 RNAs, PSF is involved in the maintenance of pools of RNA available for Rev activity. Further we show that MATR3 and PSF interact with each other and are able to associate with Rev. While Rev and PSF binds the viral pre-mRNA at the site of transcription, MATR3 interacts at the subsequent nuclear step. We propose that MATR3 and PSF define a novel pathway for RRE containing HIV-1 RNAs that is hijacked by the viral Rev protein. Furthermore, MATR3 depletion in Jurkats and a T cell line based model of HIV latency results in a drop in HIV-1 production. Intriguingly, expression of both the factors is down regulated in quiescent T cells, together with other host factors required for HIV-1 gene expression and upregulated following activation.

## Conclusions

We provide evidence that MATR3 and PSF are novel nuclear cofactors that control Rev activity. We speculate that they are the components of a nuclear pathway that is hijacked by Rev to facilitate the nuclear survival and export of intron containing viral transcripts. Our findings reveal that this mechanism may be required for efficient nuclear export of genomic viral RNA and Gag expression and may contribute to latency in resting CD4+ T lymphocytes at the post-transcriptional level.

